# A RG-II Type Polysaccharide Purified from *Aconitum coreanum* Alleviates Lipopolysaccharide-Induced Inflammation by Inhibiting the NF-κB Signal Pathway

**DOI:** 10.1371/journal.pone.0099697

**Published:** 2014-06-13

**Authors:** Xiaojun Li, Jiaye Jiang, Songshan Shi, S. W. Annie Bligh, Yuan Li, Yongbo Jiang, Dan Huang, Yan Ke, Shunchun Wang

**Affiliations:** 1 Teaching Experimental Center, Shanghai University of Traditional Chinese Medicine, Shanghai, China; 2 The MOE Key Laboratory for Standardization of Chinese Medicine, Institute of Chinese Materia Medica, Shanghai University of Traditional Chinese Medicine, Shanghai, China; 3 Shanghai R&D Center for Standardization of Chinese Medicines, Shanghai, China; 4 Department of Complementary Medicine, Faculty of Science and Technology, University of Westminster, Westminster, United Kingdom; Taipei Medical University, Taiwan

## Abstract

Korean mondshood root polysaccharides (KMPS) isolated from the root of *Aconitum coreanum* (Lévl.) Rapaics have shown anti-inflammatory activity, which is strongly influenced by their chemical structures and chain conformations. However, the mechanisms of the anti-inflammatory effect by these polysaccharides have yet to be elucidated. A RG-II polysaccharide (KMPS-2E, Mw 84.8 kDa) was isolated from KMPS and its chemical structure was characterized by FT-IR and NMR spectroscopy, gas chromatography–mass spectrometry and high-performance liquid chromatography. The backbone of KMPS-2E consisted of units of [→6) -β-D-Galp (1→3)-β-L-Rhap-(1→4)-β-D-GalpA-(1→3)-β-D-Galp-(1→] with the side chain →5)-β-D-Arap (1→3, 5)-β-D-Arap (1→ attached to the backbone through O-4 of (1→3,4)-L-Rhap. T-β-D-Galp is attached to the backbone through O-6 of (1→3,6)-β-D-Galp residues and T-β-D-Ara is connected to the end group of each chain. The anti-inflammatory effects of KMPS-2E and the underlying mechanisms using lipopolysaccharide (LPS) - stimulated RAW 264.7 macrophages and carrageenan-induced hind paw edema were investigated. KMPS-2E (50, 100 and 200 µg/mL) inhibits iNOS, TLR4, phospho-NF-κB–p65 expression, phosphor-IKK, phosphor-IκB-α expression as well as the degradation of IκB-α and the gene expression of inflammatory cytokines (TNF-α, IL-1β, iNOS and IL-6) mediated by the NF-κB signal pathways in macrophages. KMPS-2E also inhibited LPS-induced activation of NF-κB as assayed by electrophorectic mobility shift assay (EMSA) in a dose-dependent manner and it reduced NF-κB DNA binding affinity by 62.1% at 200µg/mL. In rats, KMPS-2E (200 mg/kg) can significantly inhibit carrageenan-induced paw edema as ibuprofen (200 mg/kg) within 3 h after a single oral dose. The results indicate that KMPS-2E is a promising herb-derived drug against acute inflammation.

## Introduction

Inflammation is beneficial when dealing with potential injurious agents and local tissue damage, including invading bacteria, viruses, pathogens, and damage caused by physical and chemical factors [1], [2]. However, uncontrolled inflammatory reactions can predispose to various human diseases, including cancer [3], [4]. Administering proinflammatory mediators and cytokines such as nitric oxide, TNF-α, interleukin-1β (IL-1β), and interleukin-6 (IL-6) to humans can cause redness, swelling, fever and pain as well as tissue destruction, shock and, occasionally, death [5]–[7]. Macrophages have a positive effect on resistance to various infections and cancer. They release cytokines such as IL-1β and TNF-α that are associated with both the extrinsic and intrinsic inflammatory pathways, enabling cells to secrete IL-6, interleukin-8 (IL-8), and adhesion molecules, by activating nuclear factor κB (NF-κB). NF-κB is an important transcription factor that efficiently induces expression of a variety of cytokines, adhesion molecules, and enzymes that regulate the inflammatory cascade [8], [9]. A major NF-κB activation pathway involves triggering toll-like receptors (TLRs). These TLRs act as primary sensors that detect a wide variety of microbial components and elicit innate immune responses. Upon stimulation with various TLR ligands, NF-κB is released into the nucleus and activates many proinflammatory mediators [10], [11].

Korean mondshood root is the crushed roots of *Aconitum coreanum* (Lévl.) Rapaics, a well-known traditional Chinese medicine called ‘guanbaifu’ [12]. It has been used to treat various disorders over centuries, such as cardialgia, facial distortion, epilepsy, migraine headaches, vertigo, tetanus, infantile seizures, and rheumatoid arthritis [13], [14]. Pharmacological studies have demonstrated that Korean mondshood root extract can be used to treat arrhythmias, and has analgesic and inflammatory effects [15]. Previous phytochemical studies on this herb have isolated several bioactive components, such as alkaloids, sitosterol, and organic acids [16]. The low-molecular-weight chemical constituents of *A. coreanum* have been well-investigated. However, high-molecular-weight components such as polysaccharides have rarely been explored. Research has demonstrated that oral administration of *A. coreanum* stem polysaccharides specifically inhibits tumor growth and extends the survival time of tumor-bearing mice [17]. However, no study has focused on chemical structures and anti-inflammatory activity of polysaccharides from *Aconitum coreanum* (Lèvl.). Therefore, the present study aims to isolate and analyze the chemical structure of a homogenous polysaccharide in the roots of *A. coreanum* and study its anti-inflammatory activity using LPS induced RAW 264.7 cell and carrageenan-induced hind paw edema in rats. The underlying mechanisms of the anti-inflammatory effects were explored mainly on the NF-κB signaling pathways.

## Materials and Methods

### Materials

*Aconitum coreanum* (Guanbaifu) pieces for decoction were provided by Kang Qiao Traditional Chinese Medicine Decoction Pieces Co. Ltd, (Shanghai, P. R. China). The following equipments were used: BS-100 automatic parts collection instrument (Huxi Analytical Industry Co. Ltd, Shanghai, P. R. China), Avanti TMJ-251 high-speed freezing centrifuge (Beckman Coulter Inc., Palo Alto, USA), Agilent 1100 Series HPLC system (Agilent, Wilmington, USA), Agilent 7890A/5975C-GC/MS (Agilent, Santa Clara, USA). TRIzol reagent was obtained from Invitrogen (Carlsbad, USA). The anti-TLR4 antibodies (cat. 2219S, 1∶1000), phospho-NF-κB-p65 Ser536 (cat. 3033S, 1∶1000), Phospho-IκB-α (14D4) rabbit mAb (cat. 2859, 1∶1000) and IκBα(44D4) rabbit mAb (cat. 4812, 1∶1000) were purchased from Cell Signaling Technology (Beverly, USA). Phospho-IKK was purchased from Santa Cruz Biotechnology (Santa Cruz, CA, USA). The anti-GAPDH antibodies (ab8245, 1∶5000) and the iNOS antibodies (ab9485, 1∶1000) were purchased from Abcam Inc. (Cambridge, USA). HRP-conjugated anti-mouse IgG and HRP-conjugated anti-rabbit IgG (1∶5000) were obtained from Solarbio Science & Technology Co. Ltd (Beijing, P. R. China). All other reagents were purchased from Sigma Chemical Co. (St. Louis, USA).

The mouse macrophage cell line RAW 264.7 was obtained from Shanghai Institutes for Biological Sciences (Shanghai, P. R. China) and cultured in Dulbecco's modified Eagle's medium (DMEM) supplemented with 10% FBS, 1% penicillin–streptomycin (Sigma, St. Louis, USA) at 37°C in an atmosphere of 5% (V/V) CO_2._

Eight-week-old male Wistar rats, weighting between 180 and 210 g, were purchased from Slaccas Lab Animal Ltd (Shanghai, P. R. China) (SPF II Certificate: No. SCXK 2007-0005). The experimental protocol was approved by the Center for Animal Experiments, Shanghai University of Traditional Chinese Medicine (Shanghai, P. R. China) (No. SYXK 2009-0069). These rats were housed in a temperature-controlled environment with standard rodent chow and water, under a reversed 12 h light/dark schedule. Cages were cleaned once a week, with rats handled by the tail by experienced animal care staff to transfer them between cages. Rats were euthanized using carbon dioxide after the end of the experiment. All efforts were made to minimize suffering.

### Methods

#### Extraction of Korean mondshood root polysaccharides, KMPS

Korean mondshood root pieces for decoction (10 kg) were extracted with petroleum ether at 60°C for 2 h to remove protein using a reflux condensation device. The residues were extracted twice with 15 L of distilled water at 90°C for 4 h. After centrifugation at 4000 rpm for 10 min, the supernatant was concentrated by rotary evaporation at 60°C. Four volumes of 95% EtOH were added to the concentrated solution. The sample solution was precipitated overnight in a refrigerator (4°C), and centrifuged at 4000 rpm for 10 min. The precipitate was freeze-dried and dried crude KMPS polysaccharides were obtained.

#### Isolation and purification of KMPS-2E

KMPS (10 g) was dissolved twice in distilled water in a 60°C water bath and centrifuged at 12000 rpm for 10 min. The supernatant was fractionated on a DEAE–cellulose column (50 cm × 5 cm, Cl-form), eluted stepwise with distilled water, followed by 0.2 M and 0.4 M NaCl solution, and monitored using the phenol–sulfuric acid method [18]. The fraction was collected and concentrated based on the characteristics of its peak. The sample was dialyzed with flowing water and freeze-dried using a lyophilizer (Labconco, Kansas City, USA). Then, the sample was weighed and stored. The fraction eluted with 0.2 M NaCl was further separated via gel filtration using a Superdex-200 column (100 cm × 2.5 cm), eluted with 0.2 M NaCl solution, and monitored using an RI-102 refractive index detector (Lihui Biological Technology Co., Ltd., Suzhou, P.R. China). The fractions were collected, concentrated, dialyzed against flowing water, and lyophilized to obtain the KMPS-2E powder.

#### Determination of the molecular weight

The molecular weight of the KMPS-2E was estimated through high performance gel permeation chromatography (HPGPC) using series-connected serial columns of KS-804 and KS-805. Shedex sugar packed columns were eluted with a mobile phase of 0.2 M NaCl at a flow rate of 0.8 mL/min. To estimate the molecular weight, Shedex packed columns were calibrated using standard P-series dextrans (P-5, P-10, P-20, P-50, P-100, P-200, P-400 and P-800). The column temperature was maintained at 40.0±0.1°C. A 2 mg sample of the KMPS-2E was dissolved in 1 mL mobile phase and 20 µL of the solution was analyzed in each run under the same experimental condition. The retention time was plotted against the average molecular mass of the dextrans, and from this plot the molecular mass of the sample was calculated [19].

#### Monosaccharide composition analysis

KMPS-2E (2 mg) was hydrolyzed with 2 M trifluoroacetic acid (TFA, 2 mL) at 120°C for 2 h in a sealed test tube. The TFA was removed under reduced pressure through repeated evaporation with MeOH. The hydrolyzate was successively reduced with sodium borohydride, acetylated with Ac_2_O at 100°C for 1 h, and the resulting alditol acetates were examined via GC-MS [20].

#### Reduction of uronic acid

A 200 mg sample of KMPS-2E was dissolved in 20 mL of distilled water and then 100 mg of CMC was added. The pH of the reaction mixture was maintained at 4.75 during the 3 h reaction. Once hydrogen ion uptake ceased, 2 M aqueous sodium borohydride solution was added dropwise. The pH of the mixture was maintained at 7.0 with 3 M HCl. A total of 200 mL of the borohydride solution had been added into the mixed liquor during a 2 h period [21].

#### Determination of Absolute Configuration

The reagents of A solution [(s) -(+)-1-amino-2-propanol: absolute methanol  =  1∶8], B solution (3% NaBH_3_CN m/v) and C solution (glacial acetic acid: absolute methanol  =  1∶4) were prepared. A 1.0 mg portion of the polysaccharide was dissolved in TFA (2 M, 2 mL) and the mixed solution was hydrolyzed at 120°C for 1.5 h, the hydrolysate was evaporated to dryness, and then the residue was placed into a P_2_O_5_ vacuum dryer for overnight. Then 20 µL of the residue was added into the A, B and C solutions. The mixed solution was allowed to react in an air-tight container at 65°C for 1.5 h and then evaporated to dryness. After drying, the residue was vacuum dried overnight. Then, 20 µL of pyridine and 20 µL of acetic anhydride were added and the mixture was allowed to react at 100°C for 1 h. Distilled water (1 mL) was added to the reaction liquid, and the sample solution was extracted with 2 mL of chloroform. The chloroform layer was then washed thrice with Na_2_CO_3_ solution and distilled water. The sample was dried with anhydrous sodium sulfate and analyzed by GC-MS [22], [23].

#### Methylation analysis

A modified Hakomori method [24] was adopted for the methylation analysis. Initially, dimsyl sodium (SMSM) solution was prepared for Need's method [25]. After that, the vacuum-dried polysaccharide (10 mg) was methylated three times with minor modifications. The polysaccharide was weighed precisely and dissolved in 1.0 mL of DMSO. Then, 1.0 mL of SMSM was added under water-free conditions. After incubation with stirring for 12 h at room temperature, 1 mL of iodomethane was added slowly into the polysaccharide mixture. The polysaccharide mixture was incubated in the dark for 4 h and dialyzed against flowing water for 24 h. The methylated polysaccharide was extracted three times with 2 mL of chloroform and dried under reduced pressure on a rotary evaporator. The completeness of methylation was confirmed by the disappearance of the hydroxyl absorption in the IR spectrum (Nujol). Methylated alditol acetates were prepared and analyzed by GC–MS [26].

#### Two-step partial acid hydrolysis

KMPS-2E (300 mg) was dissolved in 10 mL of 0.1 M TFA at 70°C for 2 h. The hydrolysate solution was evaporated to remove TFA through repeated evaporation with MeOH under reduced pressure. The product was lyophilized and dissolved in 2 mL of 0.2 M NaCl. The mixture was separated via gel filtration on a column (100 cm × 2.5 cm) of Superdex-75 and monitored using an RI-102 refractive index detector. The fraction was collected and concentrated based on its characteristic peak. All samples were desalted via gel filtration on a Superdex-10 column (20 cm × 1.5 cm). The degraded polysaccharide KMPS-2Ea (yield, 40% from KMPS-2E) was prepared for NMR spectroscopy. A series of oligosaccharides were prepared for mass spectrometry. The degraded polysaccharide KMPS-2Ea was further hydrolyzed with 0.2 M TFA at 70°C for 2 h. The degraded KMPS-2Eb (yield, 35% from KMPS-2Ea) was collected and the mixture was treated as described above [27].

#### NMR spectroscopy

NMR spectra were recorded using a Bruker-AVANCE 400 (Bruker BioSpin, Rheinstetten, Germany). The polysaccharide sample (40 mg) was deuterium-exchanged and dissolved in 400 µL of D_2_O (99.9% D). With acetone as the internal standard (δ_H_ 2.225 ppm, δ_C_ 31.45 ppm), the NMR spectra were recorded based on the data from the experiments (^1^H, ^13^C, HSQC and COSY). The 2D NMR spectra were obtained using the standard Bruker software, and Bruker TopSpin program was used to acquire and process the NMR data.

#### Mass spectrometry

The molecular weights of a series of oligosaccharides were determined via matrix-assisted laser desorption ionization time of flight mass spectrometry (MALDI-TOF/MS), which was carried out on a MALDI Micro MX mass spectrometer (Waters/Micromass, Manchester, UK). Solutions of the oligosaccharides (100 µg) in 1 mL of ultrapure water were infused into the electrospray source at 20 µL/min. At least twenty scans (100 to 2000 amu) were performed and averaged. This instrument was operated in a linear mode with an N_2_ laser source (337 nm), negative and positive ion detection. A minimum of twenty scans (100 to 2000 amu) were performed and averaged. Selection of a stable signal was carried out using electrospray ion trap mass spectrometry (ESI-MS).

#### Infrared spectroscopy analysis

Infrared spectra of KMPS-2E and its methylated polysaccharide were recorded as KBr-pellets [28].

#### MTT assay

RAW 264.7 cells (10^5^ cells/mL) were plated in 100 µL of DMEM per well in 96-well plates with 50, 100, 200 µg/mL of KMPS-2E and incubated for 24 h at 37°C [29]. Subsequently, the cells were incubated for 4 h with 20 µL of 3-(4,5-dimethylthiaozle-2-yl)-2,5-diphenyltetrazolium bromide (MTT) per well. The formazan produced was dissolved in 20 µL of dimethyl sulfoxide, and the optical density (OD) of each well was determined at a wavelength of 570 nm using an ELISA microplate reader.

#### Nitrite assay

RAW 264.7 cells were cultured at 37°C and 5% CO_2_. The cells (10^5^ cells/mL) were seeded to confluence in 96-well plates and cultured in serum-free medium for 12 h. The cells were pretreated for 1 h with various concentrations of KMPS-2E and then incubated for 24 h with LPS (1 µg/mL). After incubation, the nitrite concentrations of the supernatants (100 µL/well) were measured using Griess reagent [30]. The optical density at 540 nm was measured on an ELISA microplate reader. The nitrite concentration was calculated by comparison with the absorbance at 540 nm of standard sodium nitrite solutions in culture medium.

#### Western blot analysis

RAW 264.7 cells were preincubated for 6 h in serum-free medium for 12 h. The cells were pretreated with various concentrations of KMPS-2E for 3 h and then stimulated for 1 h and 24 h with and without LPS (1 µg/mL). The cells were collected and washed four times with ice-cold phosphate-buffered saline (PBS). Then, the cell lysates were treated with RIPA and PMSF (Beyotime, Haimen, P.R. China) for 1 min on ice. The cell lysates were centrifuged at 12,000 rpm for 10 min at 4°C, the protein contents in the supernatant were measured using Coomassie brilliant blue method. The lysate containing 40 µg of protein was subjected to electrophoresis on 10% sodium dodecyl sulfate-polyacrylamide gel, and the gel was transferred onto a nitrocellulose membrane. The nitrocellulose membranes were blocked for 60 min with 5% non-fat dry milk in TBS buffer containing 0.1% Tween-20 (TBST) at 25°C. The membrane was then incubated overnight with specific primary rabbit polyclonal anti-rabbit iNOS Ab (LPS pretreatment for 24 h), anti-rabbit phospho-NF-κB-p65 Ab (LPS pretreatment for 1 h), anti-rabbit phospho-IκB-α Ab (LPS pretreatment for 30 min), anti-rabbit IκB-α Ab (LPS pretreatment for 30 min) or mouse monoclonal anti-rabbit GAPDH. The membranes were washed three times with TBST and incubated for 60 min with HRP-conjugated goat anti-rabbit IgG secondary antibodies in TBST containing 5% non-fat dry milk at 25°C. After washing three times, the signals were developed using an ECL western blot detection kit and exposed to X-ray film.

#### Real-time reverse transcription–polymerase chain reaction assay

RAW 264.7 cells were cultured at 37°C and 5% CO_2_. The cells (10^5^ cells/mL) were seeded to confluence in 100-mm^2^ culture dishes and cultured in serum-free medium for 12 h. RAW 264.7 cells were treated with KMPS-2E (50, 100, and 200 µg/mL) for 1 h, and then stimulated for 24 h with and without LPS (1 µg/mL). Total mRNA was isolated from the cells using TRIzol reagent to generate cDNA for amplification [31]. The synthesized cDNA was used immediately for real-time PCR amplification using primers specific for iNOS (sense, 5′-ATC CCg AAA CgC TAC ACT T-3′ antisense, 5′-Cgg CTg gAC TTC TCA CTC-3′), IL-1 (sense, 5′-CAC CTC TCA AgC AgA gCA CAg-3′, antisense, 5′-ggg TTC CAT ggT gAA gTC AAC-3′), IL-6 (sense, 5′-TCC TAC CCC AAC TTC CAA TgC TC-3′, antisense, 5′-TTg gAT ggT CTT ggT CCT TAg CC-3′), TNF-α (sense, 5′-CCA ggA gAA AgT CAg CCT CCT-3′,antisense, 5′-TCA TAC CAg ggC TTg AgC TCA-3′) and the β-actin control (sense, 5′-AgA TgA CCC AgA TCA TgT TTg AgA-3′, antisense, 5′-ACC AgA ggC ATA CAg ggA CAA-3′). All PCR products were fractionated via electrophoresis on a 1% agarose gel, visualized with ethidium bromide, and analyzed using a Tanon GIS-2009 Gel Image system (Tanon, Shanghai, China).

#### Electrophoretic mobility shift assay

Activation of NF-κB was investigated by electrophoretic mobility shift assay (EMSA). Briefly, RAW264.7 macrophages were pre-incubated with KMPS-2E for 3 h, followed by adding LPS (1 µg/mL) and left incubation for 1 h. Nuclear extracts were prepared from LPS-treated cells using nuclear protein extraction kits(Beyotime, Jiangsu, China). Biotinylated probes for NF-κB (5”-AGT TGA GGG GAC TTT CCC AGG C-3”and 3”-TCA ACT CCC CTG AAA GGG TCC G-5”) were synthesized. EMSA assays of NF-κB activation were carried out using Chemiluminescent EMSA Kit (Beyotime) following the manufacturer's instructions.

#### Assay of anti-inflammatory activity *in vivo*

Anti-inflammatory activity was evaluated based on inhibition of carrageenan-induced hind paw edema. Fifty Wistar rats weighing 180 g to 210 g were divided into five groups (ten rats per group). Group 1 was orally given 1 mL of normal saline. Group 2 was given oral ibuprofen at 200 mg/kg. Group 3, Group 4, and Group 5 were given KMPS-2E orally at 200, 400, and 800 mg/kg, respectively. The controls were given normal saline. The treatments were given 1 h before administering the edema-inducing agent (0.1 mL of 1% carrageenan suspension with 0.9% NaCl), which was injected into the plantar surface of the left hind paw of the male rats. The volumes of the injected paws were measured at 1, 2, 3, 4 and 5 h after inducing inflammation. Edema was defined as the increase in paw volume caused by carrageenan injection. The edema and inhibition rates were calculated according to the equation in the literature [32].

## Results

### Isolation, purification and structural analysis of KMPS-2E

**Figure 1:**
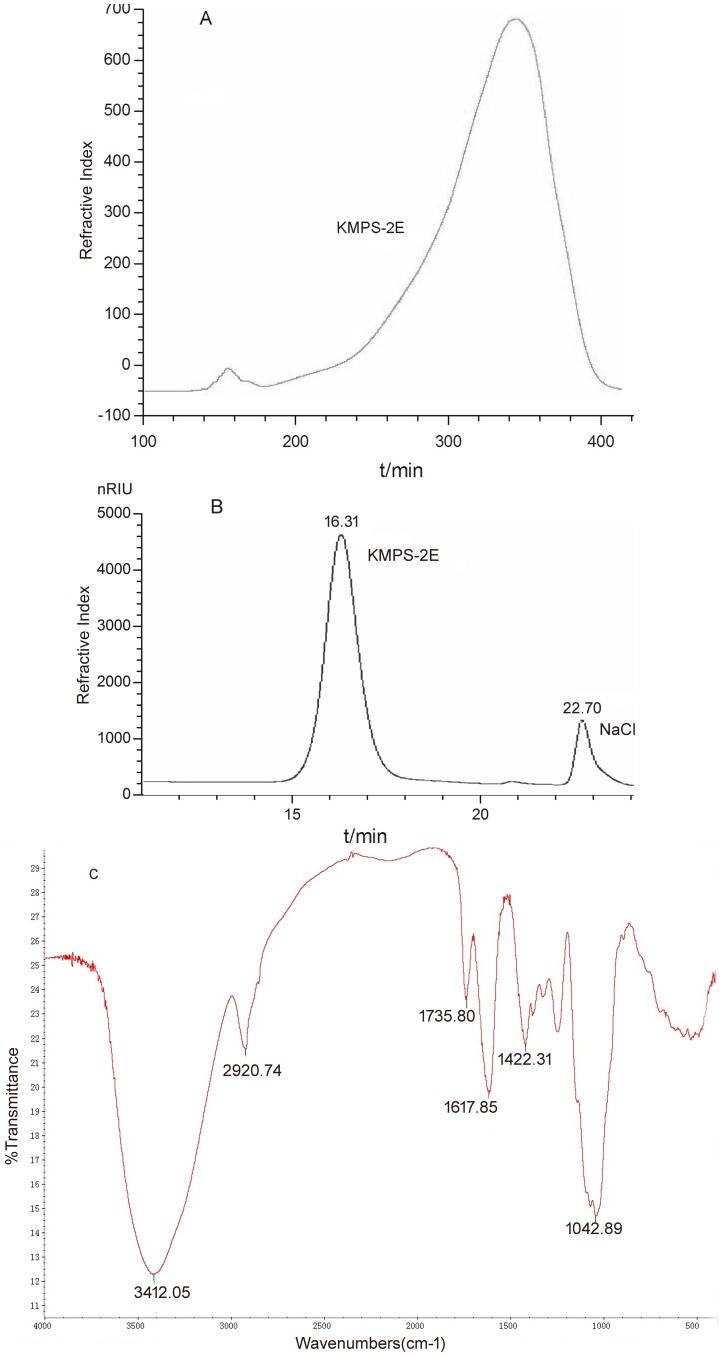
Fractionation of KMPS-2E. (A) The major fraction (KMPS-2E, t = 300∼360 min) with 0.2 M NaCl was collected for purification using Superdex 200 gel filtration chromatography; (B) the homogeneous purified KMPS-2E polysaccharide at t = 16.31 min. Its molecular weight was approximately 84,802 Da by HPGPC; (C) Infrared spectrum of KMPS-2E.

The crude polysaccharides KMPS gained from *Aconitum coreanum* (Levl.) Rapaics were separated using DEAE-32. The water, 0.2 M NaCl, and 0.4 M NaCl fractions were collected and dried under vacuum conditions. The major fraction (0.2 M NaCl) was selected for purification using Superdex 200 gel filtration chromatography (KMPS-2E, Figure 1A). The polysaccharide, KMPS-2E, was shown to be homogeneous by HPGPC (Figure 1B) and its molecular weight was approximately 84,802 Da.

### FT-IR spectral analysis

In the FT-IR spectrum of KMPS-2E, the absorbance of a broad peak at 3412.05 cm^−1^ was assigned to -OH stretching vibration (Figure 1C). The peaks at 2920.74 and 1042.89 cm^−1^ were corresponded to the C-H and C-O stretching vibration, respectively. The absorbance of the bands at 1617 and 1422 cm^−1^ are attributed to asymmetrical and symmetrical COO− stretching vibrations. A peak observed at 1735 cm^−1^ originated from the C = O stretching vibration confirming the presence of COOH (uronic acid group) in the KMPS-2E.

### Monosaccharide composition analysis and the determination of absolute configuration

The protein content of KMPS-2E was measured via the Coomassie brilliant blue method. The result showed that KMPS-2E was protein free. The monosaccharide composition of KMPS-2E including l-rhamnose, d-arabinose, and d-galactose, was detected by gas chromatography (GC) in a molar ratio of 4.82∶72.89∶22.29 (Table 1). The results also showed a small amount of uronic acid. After the reduction of the polysaccharide, the monosaccharide composition of KMPS-2E included l-rhamnose, d-arabinose, and d-galactose with a molar ratio of 7.42∶64.46∶28.12, as detected by gas chromatography (GC) showing an increase of the molar ratio of d-galactose. These findings suggested that the polysaccharide contained galacturonic acid and the molar ratio of galactose and galacturonic acid were 22.29∶5.83.

**Table 1:** Molar ratio of monosaccharide composition of KMPS-2E after different reactions.

Monosaccharide	Molar ratio of polysaccharide (%)	Molar ratio after the reduction of polysaccharide (%)	Molar ratio after first partial acid hydrolysis of polysaccharide (%)	Molar ratio after second partial acid hydrolysis of polysaccharide (%)
l-Rhamnose	4.82	7.42	12.85	15.47
d-Arabinose	72.89	64.46	26.84	3.73
d-Galactose	22.29	28.12	60.31	80.8

### Methylation analysis and partial acid hydrolysis

KMPS-2E was subjected to methylation analysis to determine the linkage types. The uronic acid in the polysaccharide was reduced before methylation analysis. The results of the methylation analysis are shown in Table 2. The GC–MS results indicated that KMPS-2E contained backbone and branched chains. In addition, the results in the Table 2 also show the linkages of partial acid hydrolysis products, and the molar ratios of terminal Ara (T-Ara), 1,5-linked Ara, and 1,3,5-linked Ara are reduced, and, 1,3,4-linked Rha, 1,4-linked Gal, 1,6-linked Gal, and 1,3,6-linked Gal are increased. We could infer that the branch chains are mainly Ara (T-Ara), 1,5-linked Ara and 1,3,5-linked Ara. This conclusion was further validated by 1D and 2D NMR data. The residual 1,4-linked Gal was obtained from reduction of 1,4-GalA based on the molar ratio of galactose and galacturonic acid in the monosaccharide composition analysis and the molar ratio of 1,4-Gal in the methylation analysis. The residual 1,3,6-linked Gal, terminal Ara (Tara), 1,5-linked Ara, and 1,3,5-linked Ara disappeared based on the molar ratios of second step partial acid hydrolysis. The molar ratio of 1,3,4-linked Rha also nearly disappeared. Conversely, the molar ratios of 1,4-linked GalA and 1,6-linked Gal increased, and the residue of 1,3,4-linked Rha increased. Therefore, the backbone was composed of GalA, Gal and Rha residues, a RG-II polysaccharide.

**Table 2:** Methylation of KMPS-2E before and after partial acid hydrolysis.

Peak No.	Methylation sugars	Linkages	Molar ratio (%)	Molar ratio of first step partial acid hydrolysis (%)	Retention time
Residue-A	2,3,5-Me3-Araf	Araf-(1→	20.04	15.55	11.705
Residue-B	2,5-Me2-Rhap	→3,4)-Rhaf-(1→	2.85	8.29	15.288
Residue-C	2,3-Me2-Araf	→5)-Araf-(1→	30.92	6.58	16.583
Residue-D	4-Me1-Araf	Gal-(1→	2.55	5.15	19.255
Residue-E	2,3,4,5-Me5-Galp	→3,5)-Araf-(1→	36.18	8.74	20.01
Residue-F	2,3,6-Me3-Galp	→4)-Galp-(1→	2.71	20.44	22.89
Residue-G	2,3,4-Me3-Galp	→6)-Galp-(1→	2.16	24.88	23.69
Residue-H	2,4-Me2-Galp	→3,6)-Galp-(1→	2.59	10.36	25.294

### NMR spectroscopy and Mass spectrometry

**Figure 2:**
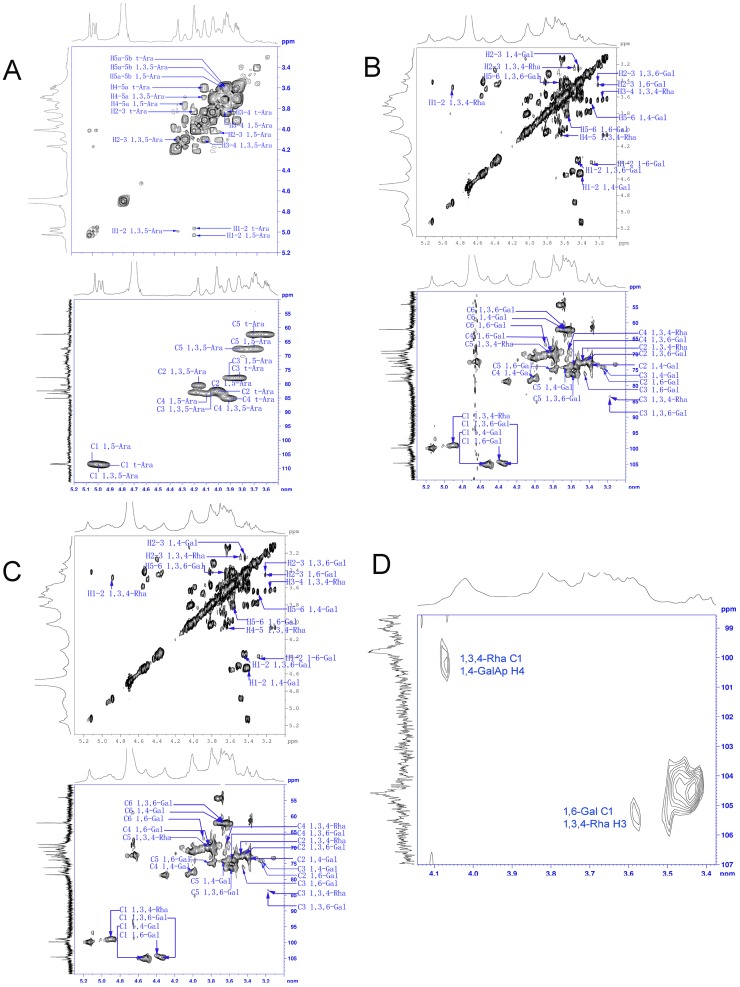
NMR spectra of KMPS-2E and its products at various acid hydrolysis steps. HSQC and H-H COSY of (A) KMPS-2E; (B) after first-step acid partial hydrolysis; (C) after second-step acid partial hydrolysis. HMBC of KMPS-2E (D) after second-step acid partial hydrolysis.

**Figure 3:**
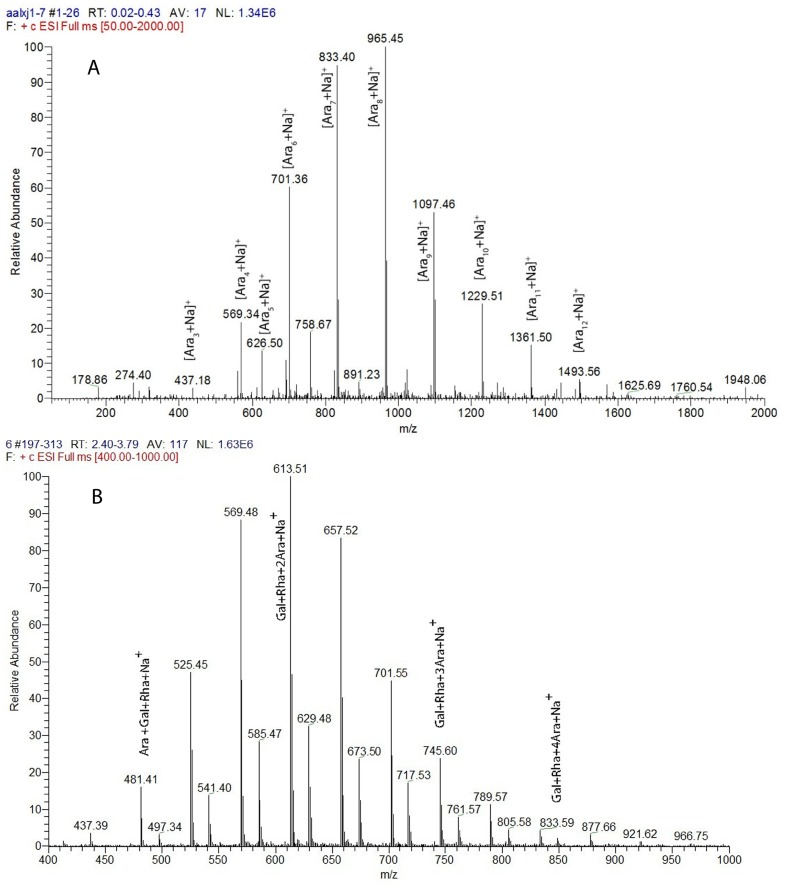
Assay of ESI-MS. (A) ESI-MS of oligosaccharides after first-step acid partial hydrolysis; (B) ESI-MS of oligosaccharides after second step acid partial hydrolysis.

The anomeric signals in the ^1^H and ^13^C NMR spectra of KMPS-2E were assigned according to sugar composition and data reported in the literature [33]–[35]. The results are shown in Table 3. The signal at *δ_H_* 4.96 was assigned to the non-reducing terminal T-Araf, and the signals at *δ_H_* 5.03 and *δ_H_* 4.99 originated from 1,5-linked Araf and 1,3,5-linked Araf, respectively. The signal at *δ_H_* 4.37 was assigned to the -1,4-linked GalpA, whereas those at *δ_H_* δ4.40 and 4.54 were assigned to -1,6-and 1,3,6-linked Galp. Finally, the signal at *δ_H_* 5.20 was assigned to 1,3,4-linked Rhap. The C-6 signals of the presence of 1,4-linked GalA were at *δ_C_* 175.96, whereas the methyl carbon signal was at *δ_C_* 54.14. The assignments of other resonances are presented in Figure 2. The anomeric signals in the ^13^C NMR spectrum of KMPS-2E were assigned mainly according to their correlations in the HSQC and H-H COSY 2D-NMR spectra. The NMR spectrum of d-arabinose was observed in the spectrum before acid hydrolysis (Figure 2A). The NMR spectrum of l-rhamnose and d-galactose were observed in the spectrum after first-step acid hydrolysis (Figure 2B). The ^13^C NMR spectrum before acid hydrolysis was compared with that after the first-step acid hydrolysis. The anomeric signals of d-arabinose weakened whereas those of l-rhamnose and d-galactose were enhanced in the ^13^C NMR spectrum. The anomeric signals in the ^13^C NMR spectrum of Ara (T-Ara), 1,5-linked Ara, and 1,3,5-linked Ara disappeared after second-step acid hydrolysis (Figure 2C). It indicates that the branch chains are mainly Ara (T-Ara), 1,5-linked Ara, and 1,3,5-linked Ara. The heteronuclear multiple bond correlation (HMBC) spectrum (Figure 2D) showed a strong cross peak between C-1 of 1,6-Gal and H-3 of 1,3,4- Rha, which confirmed the presence of 1,6-Gal→1,3,4-Rha. The trans glycosidic correlation between C-1 of 1,3,4-linked Rha and H-4 of 1,4 linked GalA, confirmed the presence of 1,3,4-Rha→1,4 GalA [36]. This conclusion was further supported by ESI-MS data (Figure 3). The results of two-step partial acid hydrolysis suggested that (Table 4 and Table 5): chemical shifts C-4/H-4 of (1→3,4)-Rha residues and chemical shifts C-6/H-6 of (1→3,6)-Gal residues were lowered. The side chain was attached to the backbone through O-4 of (1→3,4) -l-Rha and O-6 of (1→3,6) -α-d-Gal residues. Their ESI-MS profiles are shown in Figure 3A. The peaks were at *m/z* 473.18 [Ara_3_+Na]^+^, *m/z* 569.34 [Ara_4_+Na]^+^, *m/z* 626.50 [Ara_5_+Na]^+^, *m/z* 701.36 [Ara_6_+Na]^+^, *m/z* 833.40 [Ara_7_+Na]^+^, *m/z* 965.45 [Ara_8_+Na]^+^, *m/z* 1097.46 [Ara_9_+Na]^+^, *m/z* 1229.51[Ara_10_+Na]^+^, *m/z* 1361.50 [Ara_11_+Na]^+^, *m/z* 1493.56 [Ara_12_+Na]^+^. The results further proved that the branch chains were mainly Ara (T-Ara), 1,5-linked Ara, and 1,3,5-linked Ara. The ESI-MS results of the oligosaccharides after second-step acid partial hydrolysis are shown in Figure 3B, with peaks at *m/z* 481.41 [Ara+Gal+Rha+Na]^+^, *m/z* 613.51 [2Ara+Gal+Rha+Na]^+^, *m/z* 745.60 [3Ara+Gal+Rha+Na]^+^, *m/z* 745.60 [4Ara+Gal+Rha+Na]^+^.

**Table 3:** Signals in the ^1^H and ^13^C NMR spectrum of KMPS-2E.

Residues		1	2	3	4	5
T-α-Araf	H	4.96	4.00	3.83	3.91	3.69(3.36)
	C	108.77	82.70	77.80	85.10	62.33
1,5-α-Araf	H	5.03	4.02	3.92	4.09	3.70(3.85)
	C	108.34	82.50	77.80	83.50	67.52
1,3,5-α-Araf	H	4.99	4.16	4.10	3.91	3.80(3.60)
	C	108.40	80.55	82.15	85.18	67.75

**Table 4:** Signals in the ^1^H and ^13^C NMR spectra of KMPS-2E after first acid partial hydrolysis.

Residues		1	2	3	4	5	6
1,3,4-β-Rhap	H	4.88	3.47	3.57	3.60	4.03	1.22(3.76)
	C	98.81	72.60	73.54	74.21	70.84	18.10
1,4-β-GalAp	H	4.54	3.42	3.25	4.03	3.65	na
	C	105.02	72.50	74.00	77.71	74.28	175.96
1,6-β-Galp	H	4.40	3.25	3.46	3.54	3.79	3.88(3.85)
	C	104.42	74.00	76.26	71.96	74.91	67.43
1,3,6-β-Galp	H	4.35	3.44	3.21	3.55	3.64	3.84(3.61)
	C	104.70	72.00	83.32	73.74	76.45	67.43

na:not assigned.

**Table 5:** Signals in the ^1^H and ^13^C NMR spectra of KMPS-2E after second acid partial hydrolysis.

Residues		1	2	3	4	5	6
1,3,4-β-Rhap	H	4.88	3.48	3.23	3.62	3.82	1.20(3.70)
	C	98.75	72.61	83.40	69.30	69.80	18.18
1,4-β-GalAp	H	4.54	3.42	3.25	4.00	3.65	na
	C	105.02	72.50	74.00	77.73	74.28	175.96
1,6-β-Galp	H	4.40	3.25	3.46	3.54	3.79	3.88(3.85)
	C	104.42	74.00	76.26	71.96	74.91	67.43
1,3,6-β-Galp	H	4.35	3.44	3.21	3.55	3.64	3.84(3.36)
	C	104.70	72.00	83.32	73.74	76.45	62.16

na:not assigned.

### MTT assay

**Figure 4:**
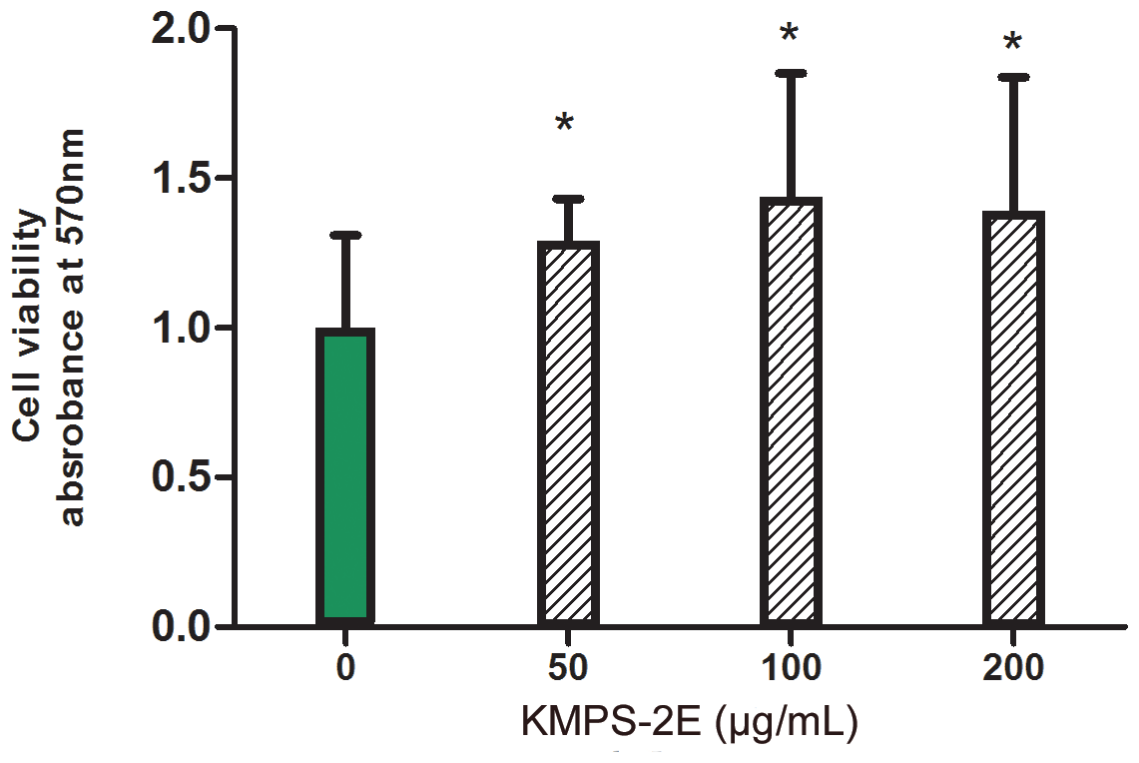
Cell viability assay and toxicity of KMPS-2E at different concentration. The samples were added to the media after the cells were maintained in fresh medium for 24± SEM, and statistical comparison was performed using an ANOVA and an LSD multiple comparisons test. *p<0.05 versus control group.

MTT assay demonstrated that KMPS-2E polysaccharide displays proliferative action on RAW 264.7 cells. When this lineage was treated with different concentration (50, 100, and 200 µg/mL) of KMPS-2E for 24 h, the cell proliferation rates were 128%, 143%, and 138%, respectively (Figure 4). These results prove that KMPS-2E is free of bacterial endotoxin. The cell proliferation assay has proved that polysaccharide possess immune-enhancing effects[37], [38]. Researchers have found that the polysaccharides from medicinal herbs can promote the expression of cell antigens on lymphocytes, stimulate T-cell proliferation, enhance secretion of a broad range of cytokines and increase serum antibody titer[39]–[41]. The lymphocyte proliferation is an important index to evaluate cellular immunity and the lymphocytes proliferation rate directly reflected the strength of cell immunity[42]. Macrophages are the first line of host defence against bacterial infection and cancer growth[43]–[45] and thus play an important role in the initiation of adaptive immune responses[46]. We can infer that KMPS-2E may have activated individual components of the immune system and promote macrophages cell proliferation.

### Nitrite assay and effect of KMPS-2E on iNOS

**Figure 5:**
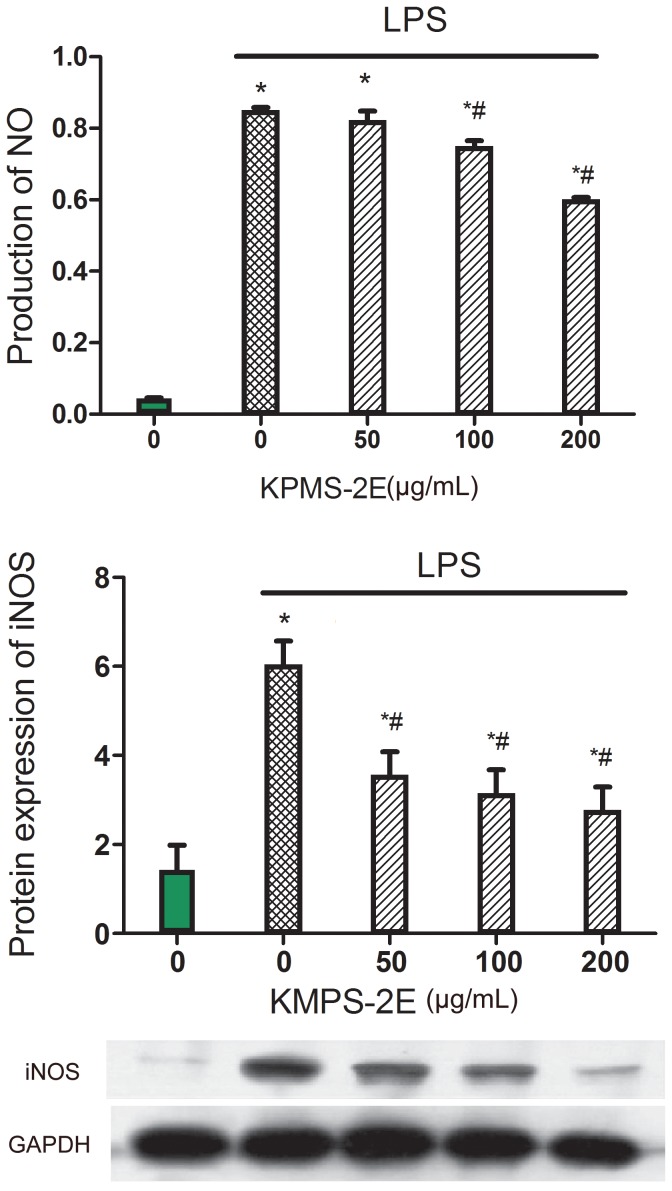
KMPS-2E inhibition of NO and iNOS. (A) KMPS-2E inhibition of NO production was detected using RAW 264.7 cells. The cells were pretreated with KMPS-2E at 50, 100, and 200 µg/mL for 1 h and then induced with LPS (1µg/mL) for 24 h. (B) Protein expression was determined using western blot analysis. Significant differences were determined using an ANOVA. *p<0.05 compared with the control group. #p<0.05 versus the model group.

The effect of KMPS-2E on NO production via an Griess reagent assay is shown in Figure 5A. The inhibitory effects of different concentrations of the polysaccharide on iNOS protein and gene expression were assessed. As shown in Figure 5B, the polysaccharide inhibited iNOS at 50 to 200 µg/mL in a concentration-dependent manner. These results demonstrate that polysaccharide inhibited iNOS expression in LPS-stimulated RAW 264.7 cells.

### Effects of KMPS-2E on the gene expression of iNOS, TNF-α, IL-1β, and IL-6

**Figure 6:**
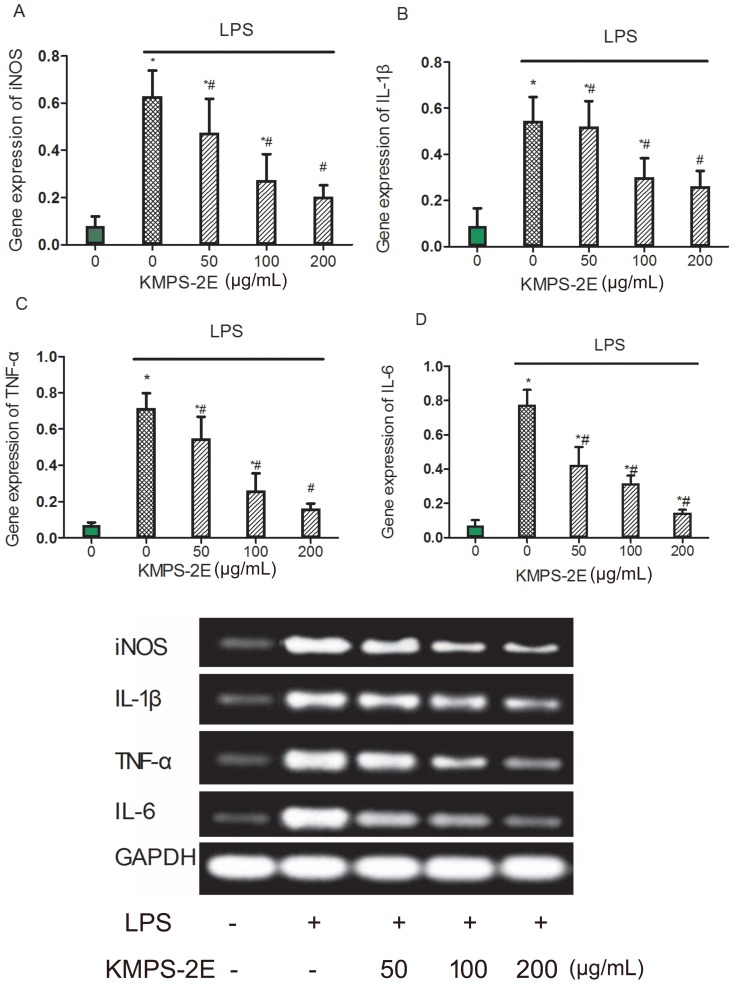
Effect of KMPS-2E on cytokine gene expression in RAW 264.7 cells. The cells were pretreated with different concentration of KMPS-2E for 1 h and treated with LPS (1µg/mL) for 24 h. Total RNA was isolated and RT-PCR was performed to examine gene expression levels of: (A) iNOS; (B) IL-1β; (C) TNF-α; (D) IL-6; (E) gene binding. After analyzing band areas using an Image Lab analysis system, target mRNA expression levels were calculated as relative ratios versus β-actin. Significant differences were determined using an ANOVA versus the model group. #p<0.05 compared with the model group. *p<0.05 compared with the control group.

Cytokines are essential molecules involved in the differentiation, maturation, and activation of cells; thus, they significantly influence the inflammatory response [47]. To determine whether KMPS-2E can affect the release of cytokines in LPS-stimulated RAW 264.7 cells, we tested the gene expression of iNOS, TNF-α, IL-1β, and IL-6. RAW 264.7 cells were pretreated with various concentrations of KMPS-2E (50, 100, and 200 µg/mL) for 1 h and stimulated with LPS (1 µg/mL) for 24 h. The total RNA was isolated from each cell preparation using TRIzol reagent. As shown in Figure 6, compared with normal group, the gene expression of iNOS (p<0.05), TNF-α (p<0.05), IL-1β (p<0.05) and IL-6 (p<0.05) were significantly increased in model group. However the level of iNOS, TNF-α, IL-1β and IL-6 were reduced in RAW 264.7 cells pretreated with KMPS-2E in a dose-dependently manner compared with the normal group.

### Effects of KMPS-2E on IκB phosphorylation and phosphorylation of IKK in LPS -stimulated RAW264.7

**Figure 7:**
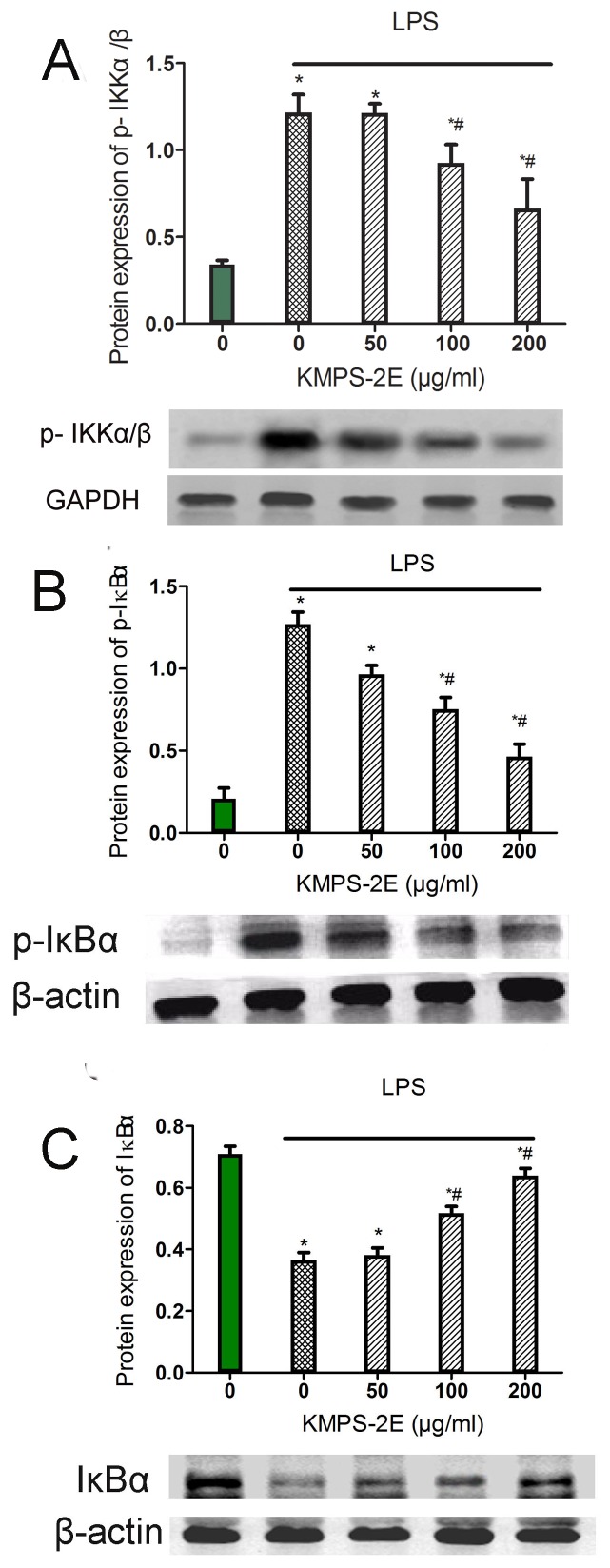
Effects of KMPS-2E on IκB phosphorylation and degradation in LPS -stimulated RAW264.7. Cells were pretreated with vehicle or KMPS-2E for 3h before treatment with the combination of LPS (1 µg/ml) for another 30 min. After treatment, Cells were then harvested, and p-IKKα/β (A), IκBα phosphorylation (B) or IκBα level (C) were determined using western blot. Data are presented as means ± S.E. (error bars). Significant differences were determined using an ANOVA versus the model group. *p<0.05 compared with the control group and #p<0.05 compared with the model group.

NF-κB activity is tightly controlled by binding to the IκB inhibitor protein, which prevents cytosolic NF-κB from entering the nucleus. Once phosphorylated by the IκB kinase (IKK) complex, IκB dissociates from the NF-κB subunits, is ubiquitinated, and is rapidly degraded by the proteasome [48]. IKK phosphorylation of the IκBα Ser32 residues was proposed as being a major mode for IκBα degradation, leading to NF-κB translocation and activation[49]. We examined the extent of IκBα phosphorylation was altered after the LPS induction. Western blot assay was shown that LPS caused significantly phosphorylation of IKKα/β and KMPS-2E pre-treatment inhibited this increase (Fig. 7A). In addition, the total amount of the IκB protein was also examined after LPS- induced for 30 min. Treatment of cells with KMPS-2E affected LPS induced IκBα phosphorylation (Fig. 7B). Compared with the normal group,LPS caused a marked increase in IκBα phosphorylation. Furthermore,KMPS-2E indeed inhibited degradation of IκBα in LPS-stimulated RAW264.7 (Fig. 7C). These results suggested that the IκBα phosphorylation and subsequent IκBα degradation in LPS-stimulated cells were inhibited by KMPS-2E on NF-κB signaling.

### KMPS-2E inhibited LPS-induced NF-κB activation in RAW 264.7 cells

**Figure 8:**
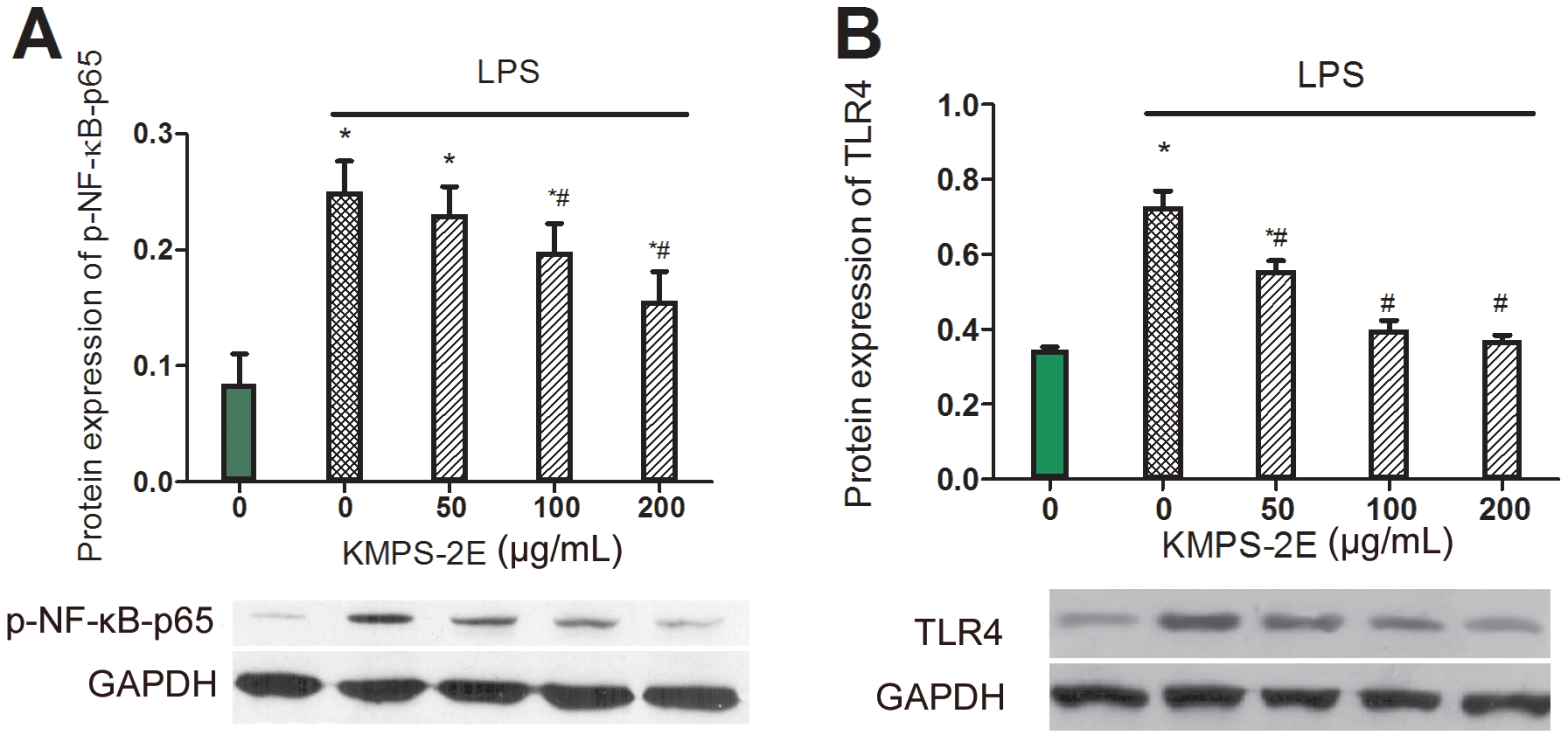
Inhibition of phospho-NF-κB–p65 and TLR4 expression. (A) The effect of KMPS-2E on phospho-NF-κB–p65 expression in RAW 264.7 cells. The cells were pretreated with 50, 100, 200 µg/mL KMPS-2E for 3 h and co-incubated with LPS (1µg/mL) for 1 h. (B) Inhibition of TLR4 by KMPS-2E in RAW 264.7 cells. RAW264.7 were pretreated with KMPS-2E at 50, 100, 200 µg/mL for 1 h and then induced with LPS (1µg/mL) for 24 h. TLR4 expression was determined using western blot analysis. Significant differences were determined using an ANOVA versus the model group. #p<0.05 compared with the model group, *p<0.05 compared to the control group.

Under unstimulated conditions, NF-κB is present in the cytoplasm, rendering it inactive. Upon extracellular stimulation, NF-κB is activated and translocates into the nucleus to regulate the synthesis and release of many proinflammatory mediators. We further studied whether TLR4 led to the activation of NF-κB signaling while the RAW 264.7 cells were stimulated with LPS. The expression of phospho-NF-κB–p65 and TLR4 were measured after LPS stimulation. The level of the phospho-NF-κB–p65 protein was increased after LPS treatment. KMPS-2E inhibited phospho-NF-κB–p65 in a dose-dependent fashion. The results showed that the model group had higher TLR4 expression than the control group, and the TLR4 secretion was dose dependent (Figure 8).

### Inhibition of LPS-induced NF-κB nuclear protein DNA binding activity by KMPS-2E

**Figure 9:**
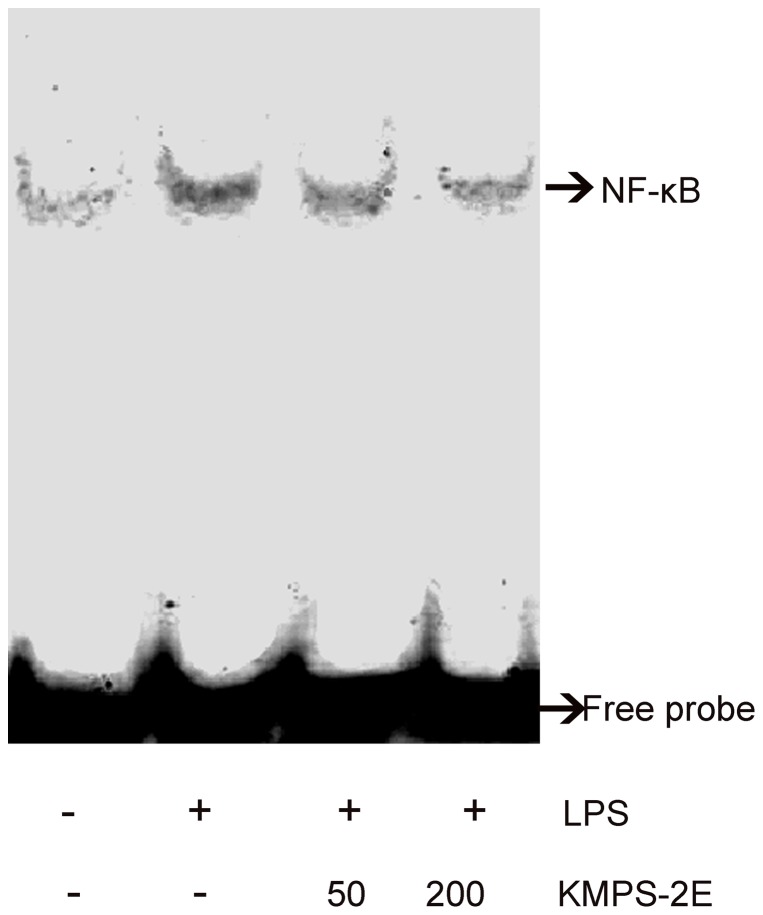
Effects of KMPS-2E on LPS-induced DNA-binding activity in RAW264.7 cells. The cells were pre-treated for 3 h with KMPS-2E and treated for 1h with LPS (1µg/ml). After incubation, the nuclear extracts were prepared from the cells and analyzed via EMSA for the activated NF-κB using radiolabeled oligonucleotide probes.

Since activation of NF-κB is responsible for the production of proinflammatory cytokines and NO upon LPS stimulation, we hypothesized that the NF-κB signaling pathway might be involved in KMPS-2E mediated inhibition of proinflammatory cytokines and iNOS expression. To further assess whether LPS-stimulated iNOS expression was associated with increased levels of nuclear NF-κB and whether KMPS-2E could inhibit NF-κB DNA-binding activity, EMSA was conducted to determine the DNA-binding activity. RAW264.7 cells were pretreated with and without KMPS-2E for 3 h, stimulated with LPS for 1 h, and then subjected to EMSA. The results showed that LPS caused a significant increase in the binding of NF-κB to DNA (Fig. 9, NF-κB-DNA in lane 2). KMPS-2E at a concentration of 200 µg/mL significantly inhibited the binding of NF-κB to biotin-labeled DNA probes (Fig. 9, lane 4), suggesting that the LPS-induced transcriptional activity of NF-κB was significantly reduced by the treatment with KMPS-2E. These results have shown that KMPS-2E has anti-inflammatory effects.

### Effect of different KMPS-2E concentrations on rat paw edema

The paw edema of Wistar rats increased progressively and KMPS-2E (200, 400, and 800 mg/kg, p.o.) exhibited a dose-dependent anti-inflammatory activity (Table 6). KMPS-2E significantly inhibited carrageenan-induced paw edema in Wistar rats at all doses employed within 4 h after treatment. KMPS-2E reduced the paw edema comparatively with ibuprofen at 200 mg/kg in the first 3 h. The highest anti-inflammatory activity of the 800 mg/kg treatment (p<0.01) was observed at 4 h after carrageenan injection comparing with ibuprofen.

**Table 6:** Effect of different KMPS-2E concentrations on rat paw edema after 1% carrageenan injection^a^.

Increase in paw volume (mL) at different intervals ± S. E and Inhibition ratio (%)						
Group	Dose (mg/kg) PO	1 h	2 h	3 h	4 h	5 h
Control	-	0.377±0.062	0.423±0.119	0.443±0.127	0.400±0.117	0.284±0.091
Ibuprofen	200	0.343±0.088	0.354±0.089	0.282±0.138*	0.205±0.064*	0.221±0.066
		9.09	16.26	36.37	48.69	22.02
Low	200	0.312±0.071	0.325±0.067	0.330±0.145	0.279±0.111*	0.307±0.126
		17.38	23.13	25.45	30.3	−8.07
Medium	400	0.311±0.082	0.310±0.106*	0.301±0.118*	0.279±0.056*	0.253±0.098
		17.71	26.78	32.02	30.07	10.97
High	800	0.300±0.105	0.283±0.083**	0.284±0.069*	0.251±0.058**	0.249±0.090
		20.51	33.1	35.92	37.26	12.43

a Values are expressed in mean±SEM (n = 10), % inhibition of paw edema is indicated in parenthesis.* p<0.05, ** p<0.01 compared with the control group (ANOVA).

## Discussion

*A. coreanum* stem polysaccharides have been reported for their inhibition in tumor growth[17]. Mounting evidence suggest that inflammation is an important risk factor for tumorigenesis. Inflammation promote tumor development not only through enhancing proliferation of tumor cell and inducing DNA damage, but also stimulates angiogenesis and tissue remodeling[50]. Anti-tumor effect of polysaccharides might be partially due to its anti-inflammatory properties. Inflammation can play an important role in tumorigenesis [50]. In this study, we investigated anti-inflammatory activity and its underlying mechanisms of an isolated RG-II type polysaccharide KMPS-2E from roots of *Aconitum coreanum* (Lévl.) Rapaics. The extensive characterization of the structure of the KMPS-2E clearly demonstrated that the backbone is composed of GalA, Gal and Rha residues with a molecular weight of 84.802 kDa.

Inducible nitric oxide synthase (iNOS) is a major inflammatory mediator that contributes to the pathogenesis of inflammation. The iNOS in macrophages is induced by LPS and consequently leads to NO overproduction, which is crucial in the pathogenesis of a variety of inflammatory diseases. NO acts as a neurotransmitter, vasodilator, and immune regulator in a variety of tissues at physiological concentrations. However, increased iNOS expression and the release of large amounts of nitric oxide can cause diseases such as rheumatoid arthritis, chronic hepatitis, and pulmonary fibrosis. Therefore, inhibiting iNOS expression in macrophages may be used to treat diseases related to increased nitric oxide production.

To identify mechanisms by which KMPS-2E inhibits iNOS activity, we determined whether NF-κB is necessary for the stimulation of iNOS and cytokines. To confirm the inhibitory effect of KMPS-2E on NF-κB activation, we examined the dose-dependent decrease in phospho-NF-κB–p65 protein in RAW 264.7 macrophage cells. We tested the effects of the polysaccharide on the NF-κB activation signal. In this study, we used LPS to activate NF-κB via TLR4. Once activated, the p65 NF-κB subunit dissociates from its inhibitory protein IkB-α and translocates from the cytoplasm to the nucleus where it triggers the transcription of specific target genes such as iNOS and IL-1β [51], [52]. The results have showed that KMPS-2E dose-dependently inhibits the LPS-induced gene expression of IL-1β, IL-6, TNF-α, and iNOS. Western blot analysis revealed that the polysaccharide inhibits LPS-induced protein expression of iNOS, phospho-NF-κB–p65, phospho-IKK and TLR4. IκBα phosphorylation and subsequent IκBα degradation in LPS induced cells were inhibited by KMPS-2E. In addition,EMSA analysis have shown that KMPS-2E inhibits NF-κB DNA-binding activity. These findings suggest that the polysaccharide inhibits the expression of inflammatory cytokines mediated by the NF-κB signal pathways.

## Conclusion

KMPS-2E, a RG-II polysaccharide, from the roots of *Aconitum coreanum* (L·vl) Rapaics has the following structure: the backbone consisted of units of [→6) -β-d-Galp (1→3)-β-l-Rhap-(1→4)-β-d-GalpA-(1→3)-β-d-Galp-(1→] with the side chain →5)-β-d-Arap (1→3, 5)-β-d-Arap (1→ attached to the backbone through O-4 of (1→3,4)-l-Rhap. T-β-d-Galp is attached to the backbone through O-6 of (1→3,6)-β-d-Galp residues and T-β-d-Ara is connected to the end group of each chain. We proved that KMPS-2E has significant anti-inflammatory effects using both in vivo and in vitro models. KMPS-2E inhibits iNOS expression in a concentration–dependent manner. It inhibits the expression of inflammatory cytokines mediated by the NF-κB signal pathways in macrophages. Moreover, KMPS-2E has no cytotoxicity up to 200 µg/mL. Therefore, a RG-II type polysaccharide, such as KMPS-2E, will contribute significantly to the development of a novel, natural anti-inflammatory agent.
